# Biomechanical evaluation of a novel L-shaped side-locking plate combined with OLIF: a finite element analysis considering 3 different bone densities

**DOI:** 10.3389/fsurg.2026.1779510

**Published:** 2026-03-20

**Authors:** Zemin Wang, Lijun Wang, Honglai Zhang, Wei Guo, Wei Yang, Wanzhong Yang, Shiyong Wang, Rong Ma, Zhaohui Ge

**Affiliations:** 1 First Clinical Medical College, Ningxia Medical University, Yinchuan, China; 2 Shandong Weigao Orthopedic Materials Co., Ltd., Weihai, China; 3 Department of Orthopedics, General Hospital of Ningxia Medical University, Yinchuan, Ningxia, China

**Keywords:** biomechanics, bone densities, finite element analysis, l-shaped side-locking plate, oblique lateral interbody fusion, supplemental fixation

## Abstract

**Background:**

Oblique lateral interbody fusion (OLIF) is a minimally invasive technique widely used for lumbar degenerative diseases. However, high rates of cage subsidence, particularly in osteoporotic bone, necessitate supplemental fixation. Traditional bilateral pedicle screw (BPS) fixation compromises OLIF's minimally invasive advantages, while conventional lateral plates provide limited stability in the sagittal plane. To address these limitations, we developed and evaluated a novel L-shaped side-locking plate (NLSLP) using finite element analysis.

**Methods:**

A validated L3-S1 finite element model was adapted to simulate L4/5 OLIF. Four surgical models were constructed: stand-alone (SA) OLIF, OLIF with a two-screw lateral plate (LP-2), OLIF with bilateral pedicle screws (BPS), and OLIF with the NLSLP. Three bone density conditions—normal bone density (NBD), osteopenia, and osteoporosis—were modeled to evaluate the biomechanical performance of each configuration. The range of motion (ROM), stress distribution in the endplates, adjacent intervertebral discs, and internal fixation were analyzed under vertical load (400 N) and torque (7.5 N·m).

**Results:**

All NLSLP configurations significantly reduced ROM at the L4-L5 segment, with lateral bending and axial rotation reductions exceeding 85%, and flexion-extension reductions of 85.84% and 75.01% for the NLSLP-d model. Compared to LP-2, the NLSLP system provided 17.85%–18.22% greater restriction in sagittal plane motion. In osteoporotic models, the NLSLP still maintained biomechanical stability, with ROM reduction exceeding 65%. Increased stress in adjacent intervertebral discs was observed in all surgical models, with the NLSLP showing a 39.86%–45.12% increase in lateral bending stress. Stress on the L5 superior endplate was reduced across all loading conditions in the NLSLP model compared to the SA model, thus reducing the risk of endplate damage and cage subsidence. Internal fixation stress remained well below the material fatigue and yield strength, indicating favorable stress distribution in NLSLP.

**Conclusions:**

The NLSLP represents a viable adjunctive fixation option for OLIF, offering superior sagittal plane stability compared to LP-2 while retaining the minimally invasive and single-position advantages of the OLIF procedure.

## 1 Introduction

Oblique lateral interbody fusion (OLIF) is a minimally invasive technique introduced by Silvestre et al. (1) for treating lumbar degenerative diseases. Unlike conventional posterior approaches such as posterior lumbar interbody fusion (PLIF) and transforaminal lumbar interbody fusion (TLIF), OLIF utilizes the anatomical corridor between the abdominal aorta and psoas major to access the disc space, thereby preserving posterior spinal structures—including laminae and facet joints—and minimizing muscle disruption (2). The lateral implantation of a large, lordotic cage achieves indirect decompression by restoring intervertebral height and tensioning the ligamentum flavum, thereby effectively correcting sagittal alignment (3). Therefore, it features minimally invasive indirect decompression, effective intervertebral fusion, and rapid postoperative recovery, which has led to its widespread recognition and adoption by spinal surgeons globally. However, cage subsidence remains a critical clinical issue. Abe et al. (4) reported a cage subsidence rate of 9.03%. Hu et al. (5) found that the cage subsidence rate reached as high as 40.34% following stand-alone OLIF procedures. This risk appears to be further intensified by the presence of osteoporosis. Our previous research also indicates that with increasing severity of osteoporosis, the maximum stress on the upper and lower endplates of the fusion segment rises significantly, thereby elevating the risk of cage subsidence (6). Cage subsidence not only results in loss of intervertebral height and recurrence of neurological deficits but also necessitates revision surgery in severe cases, imposing substantial physical distress and economic burden on patients (7). Therefore, supplementary fixation should be considered a more judicious strategy for patients at elevated risk of cage subsidence following OLIF.

Various supplemental fixation techniques have been described in the literature, such as bilateral pedicle screw (BPS) fixation, lateral plate (LP) fixation, and lateral screw-rod (LSR) fixation (8, 9). BPS fixation, although recognized as the reference standard for biomechanical stability, inevitably contradicts several fundamental advantages of OLIF: longer operative time, patient repositioning, greater soft tissue disruption, increased bleeding, higher radiation exposure, and accelerated adjacent segment degeneration risk due to excessive construct rigidity (10). Although traditional two-screw lateral plates（LP-2）can effectively harness the minimally invasive benefits of OLIF, they offer limited control in the sagittal plane. In the *in vitro* study conducted by Wang et al. (11), it was demonstrated that the LP-2 construct exhibited only a 14.8% reduction in range of motion during flexion compared to the SA fixation, suggesting comparatively diminished flexion–extension stability. Therefore, there is a pressing need for a lateral fixation system that provides robust stability in the sagittal plane while preserving the single-position, tissue-conserving advantages characteristic of OLIF.

In a comparative biomechanical study, Heller et al. (12) showed that bicortical fixation of lateral mass screws in the mid-to-lower cervical spine results in superior pull-out strength compared to unicortical fixation. Similarly, biomechanical tests by Giordano et al. (13) demonstrated that an L-shaped spatial structure, secured with three screws, enhances shear and rotational resistance at the distal end of fractures. Building on the biomechanical principle that bicortical fixation improves screw pull-out strength and that a three-screw L-shaped configuration offers greater resistance to shear and rotational forces, we developed a novel L-shaped side-locking plate (NLSLP) for use in OLIF.

We hypothesize that the NLSLP will provide superior biomechanical stability compared to traditional fixation systems like LP-2, particularly in the sagittal plane. By incorporating a three-screw L-shaped design, the NLSLP is expected to improve resistance to shear and rotational forces, offering enhanced fixation stability while preserving the minimally invasive nature of OLIF. This study aims to evaluate the biomechanical performance of NLSLP and determine its potential as an effective adjunctive fixation option for OLIF, particularly in patients with osteoporotic bone.

## 2 Materials and methods

### 2.1 Novel L-shaped side-locking plate design

**Figure 1:**
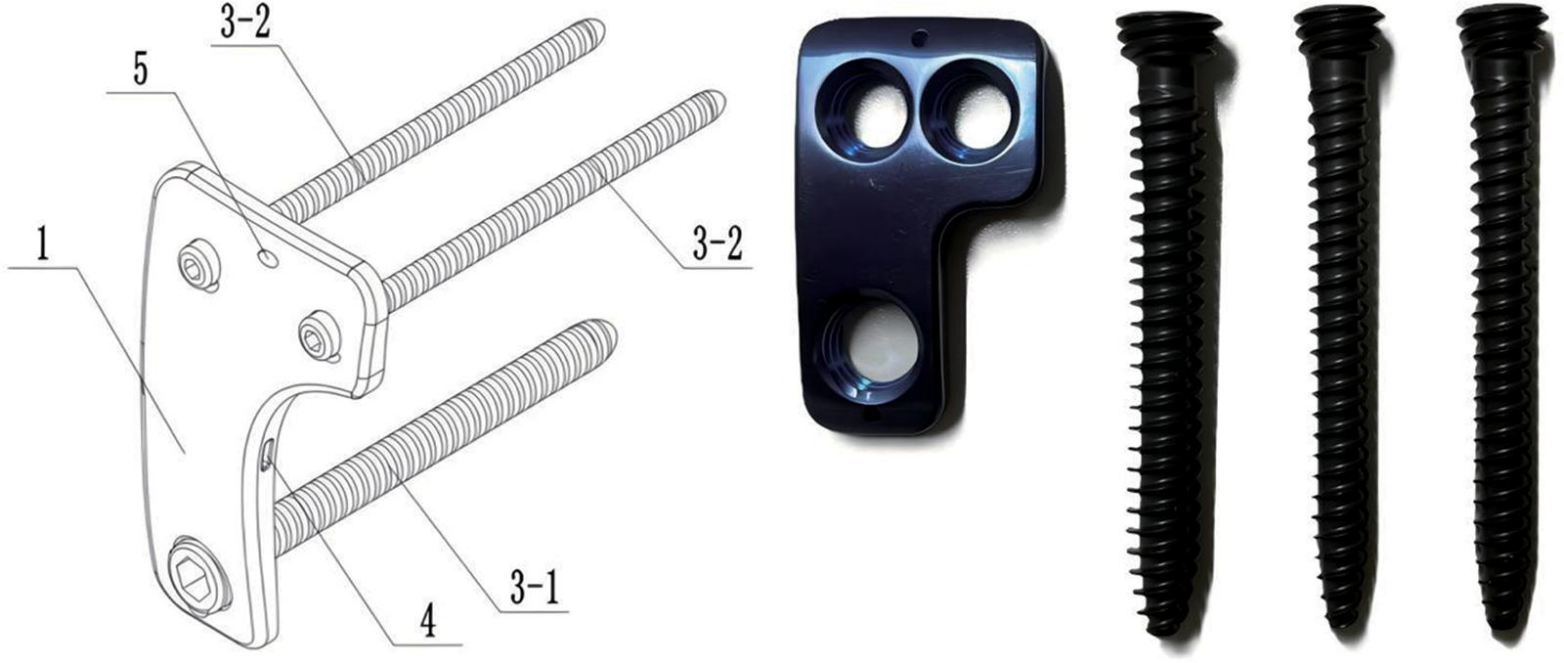
The novel L-shaped side-locking plate (NLSLP). 1. lateral plate. 3-1. Cephalad screw. 3-2. Caudal screw. 4. Grasping Card Slot. 5. Kirschner wire insertion hole.

As illustrated in Figure 1, the locking plate is designed with an L-shaped configuration, featuring a primary locking screw hole at the distal end of the longer arm and two secondary screw holes on the shorter arm. After cage implantation, the length of the lateral plate is constrained by the anatomical distance between the superior and inferior segmental arteries, while the width of the surgical corridor is crucial for determining the optimal plate width. Considering these anatomical and surgical limitations, the final dimensions of the plate were established as 40 mm in length, 24 mm in width, and 5 mm in thickness. The plate is anatomically contoured with a 30° arc in both the sagittal and coronal planes to ensure optimal fit along the lateral surface of the lumbar vertebrae. The locking screw holes have varying diameters, allowing for a more efficient screw configuration, while the reduced number of openings in the plate enhances its structural integrity and mechanical strength.

All three locking screws are fully threaded cortical bone screws. The diameter of the second locking screw ranges from 5.5 to 6.5 mm. To enhance the screw's holding strength, the diameter of the first locking screw is 1–2 mm larger than that of the second screw, as only one screw is used for fixation at the top. The screw heads penetrate the contralateral cortical bone, increasing the pull-out resistance. Additionally, the three locking screws are arranged in a triangular configuration, effectively integrating the locking plate and screws into a single unit, which improves the spatial stability of the locking screw structure.

### 2.2 Construction of the intact model of the lumbar vertebrae

**Figure 2:**
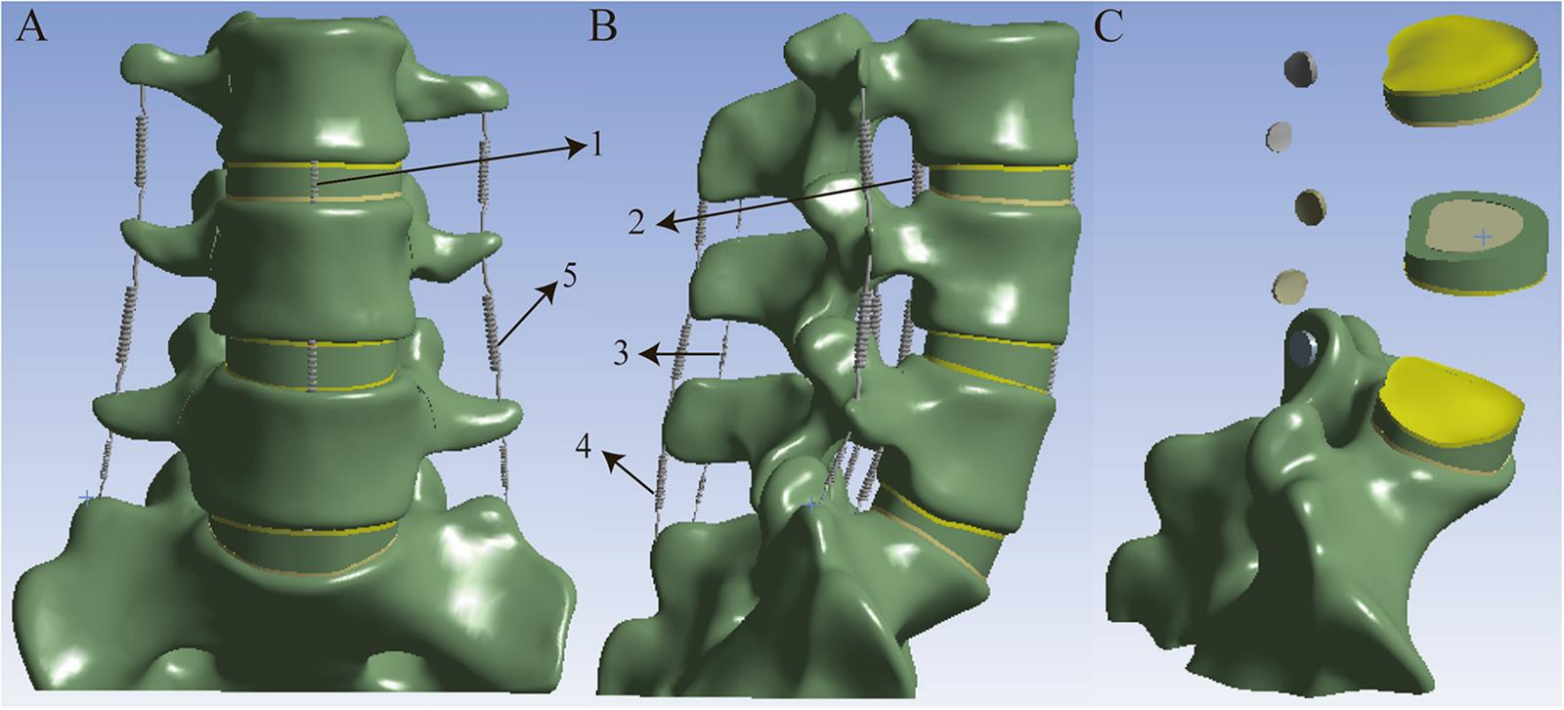
L3-S1 finite element model: **A:** frontal **B:** lateral **C:** facet joints, endplate and intervertebral disc. 1. anterior longitudinal ligament. 2.posterior longitudinal ligament. 3.interspinous ligament. 4.supraspinous ligament. 5.intertransverse ligament.

This study was approved by the Ethics Committee of the Affiliated General Hospital of Ningxia Medical University (Ethical Approval No.: 2019-308). Written informed consent was obtained from the participant. All procedures were performed in accordance with the relevant ethical guidelines and regulations. A healthy male volunteer, aged 32 years, weighing 70 kg, and 173 cm in height, with no prior history of lumbar spine disorders, was recruited for this study. CT scan data with a slice thickness of 0.625 mm were provided by the Department of Radiology at the General Hospital of Ningxia Medical University. In Mimics Research 21.0, a medical finite element modeling software, threshold segmentation and mask editing were applied to isolate the L3–S1 vertebrae for three-dimensional (3D) reconstruction. Subsequently, the 3D model generated in Mimics was imported into Geomagic Wrap 2017. To mitigate potential stress concentration issues in subsequent analyses, the model surface was systematically smoothed using a series of operations, including triangular mesh refinement, artifact removal, hole filling, feature suppression, and surface smoothing. The smoothed model was saved in STP format and imported into SolidWorks 2018. Within the Part interface, anatomical models of cortical bone, cancellous bone, posterior elements, intervertebral disc, endplate, and articular cartilage were constructed using sketching, extrusion, solid translation and duplication, and solid splitting operations. The intervertebral disc is composed of 44% nucleus pulposus and 56% annulus fibrosus (14). The cortical bone has a thickness of 1 mm, whereas the endplate is 0.5 mm thick (15). The established model was imported into ANSYS Workbench 21.0 (ANSYS, Inc., United States). In accordance with relevant literature, the following ligaments were added at their respective anatomical locations: the anterior longitudinal ligament, posterior longitudinal ligament, intertransverse ligament, ligamentum flavum, supraspinous ligament, interspinous ligament, and joint capsule ligament (16, 17), Figure 2.

### 2.3 Construction of surgical models

**Figure 3:**
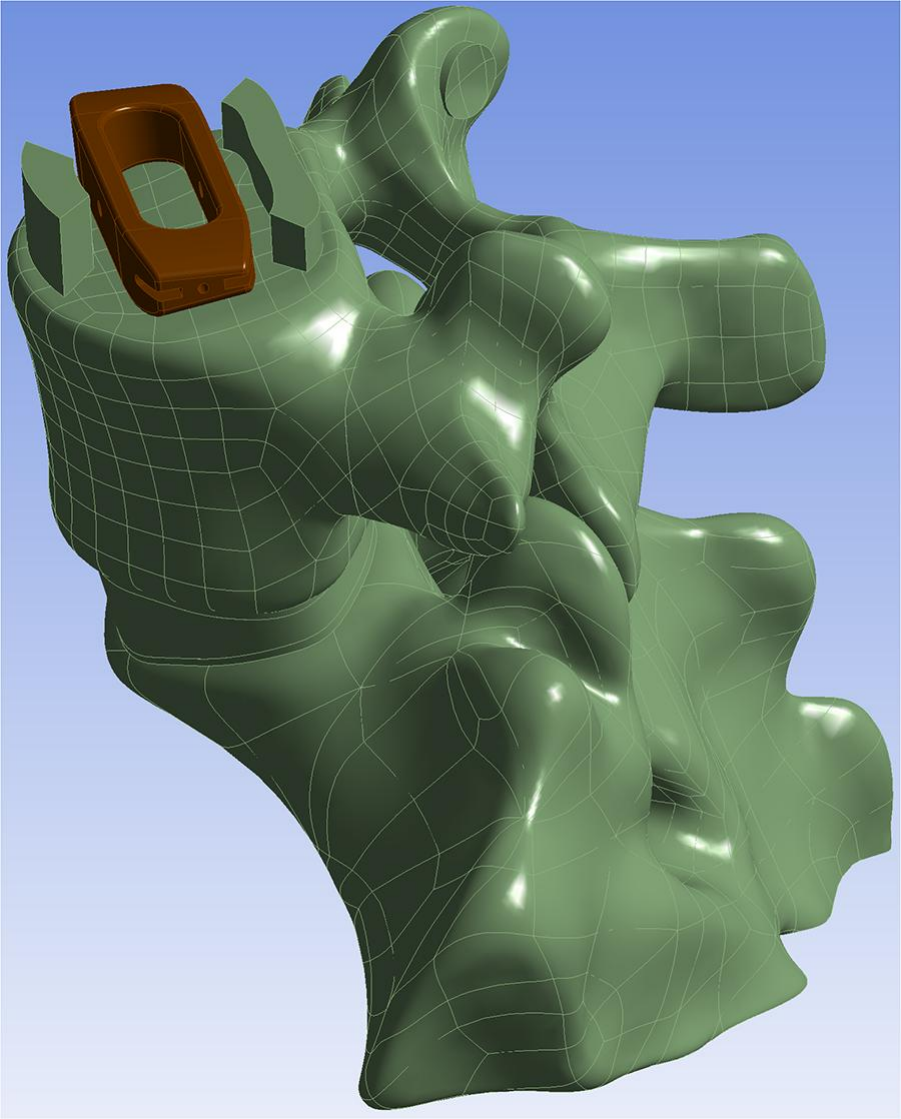
The position of the cage in the intervertebral disc.

**Figure 4:**
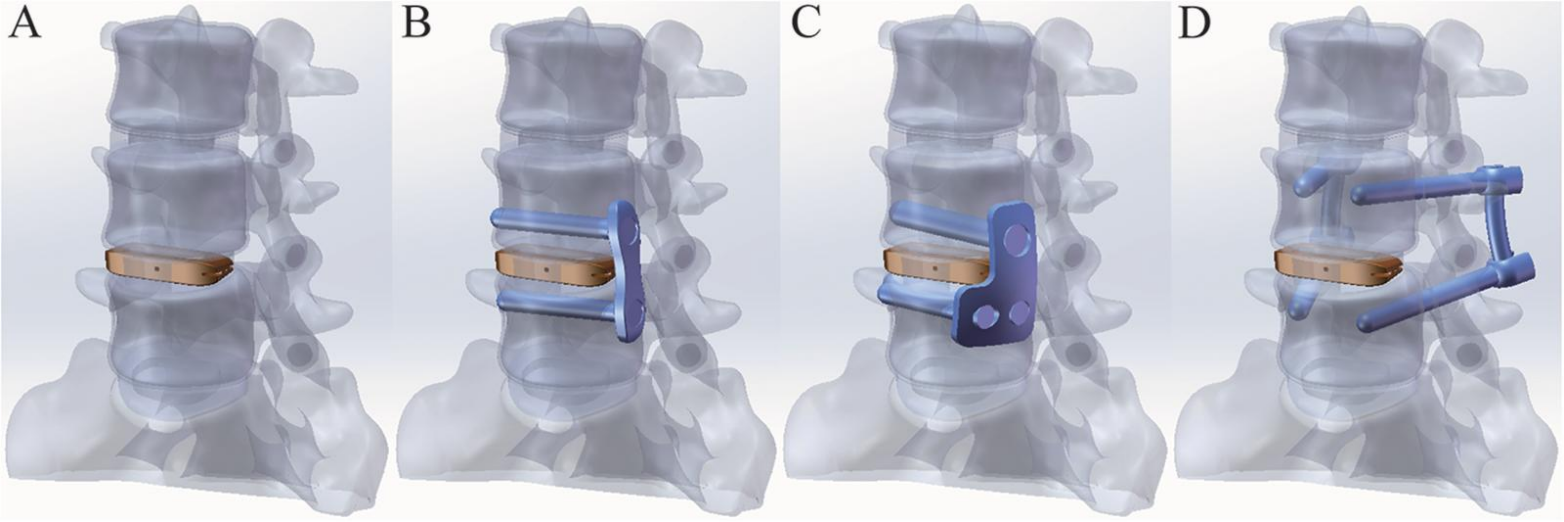
Various finite element models: **A:** OLIF Stand alone (SA) **B:** OLIF with a 2-screw lateral plate (LP-2); **C:** OLIF with novel L-shaped side-locking plate (NLSLP); **D:** OLIF with bilateral pedicle screw fixation (BPS).

**Figure 5:**
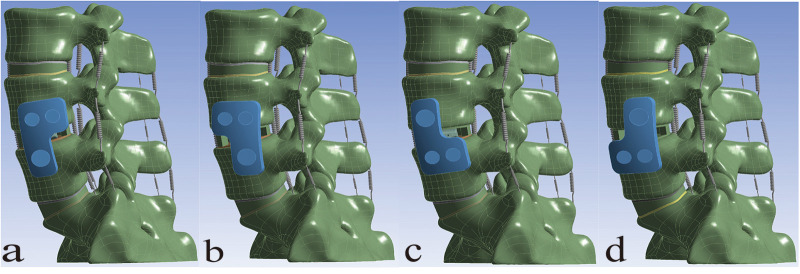
Four fixation configurations of the novel L-shaped side-locking plate (NLSLP). **(a)** “┏” orientation; **(b)** “┓” orientation; **(c)** “┗” orientation; **(d)** “┛” orientation.

The models of the cage and screws should be carefully selected based on the dimensions of the reconstructed vertebral body to ensure optimal biomechanical stability and clinical outcomes. In this study, the Clydesdale cage (Medtronic, USA) was selected, with dimensions of 45 mm in length, 18 mm in width, and 10 mm in height. The Clydesdale interbody fusion device features an arcuate design with a 6° lordotic angle between the superior and inferior surfaces. The lateral plate measures 40 mm in length and 12 mm in width, while the side screws have a diameter of 6.5 mm and a length of 50 mm. The pedicle screws are 6.0 mm in diameter and 45 mm in length, and the connecting rod has a diameter of 5.5 mm and a length of 53 mm. All internal fixation components are fabricated from titanium alloy (Ti-6Al-4 V). The L4/5 intervertebral space was chosen as the surgical segment, where partial annulus fibrosus and nucleus pulposus were resected, and a fusion device was implanted at the middle portion of the disc space to construct the stand-alone OLIF (SA OLIF) model (Figure 3). Based on this, the following constructs were developed: OLIF with a two-screw lateral plate (LP-2), OLIF with a novel L-shaped side-locking plate (NLSLP), and OLIF with bilateral pedicle screw fixation (BPS) (Figure 4). Four versions of the NLSLP model were created, each with a different installation position on the lateral side of the vertebral body (Figure 5).

### 2.4 The material properties, loading conditions, and boundary constraints of the model

Based on prior literature, the material properties of cortical bone, cancellous bone, posterior structures, articular cartilage, endplates, intervertebral discs, cages, and internal fixation devices were defined accordingly (18, 19) (Table 1). The osteoporosis model is defined as follows: the elastic modulus of all bone structures is reduced, with cancellous bone decreased by 66%, cortical bone, endplates, and posterior elements reduced by 33%, while the properties of other structures remain unchanged (20). Since osteopenia is characterized by a continuous range of T-scores, its model cannot simulate a continuous variable. Therefore, the average elastic modulus of normal bone and osteoporotic bone was used to represent osteopenia (6). Ligaments are modeled as spring elements that can withstand tensile forces but do not resist compressive loads (21). Different Young's Modulus were assigned to cortical and cancellous bone to sequentially establish the normal bone density model (OLIF-SA 1, OLIF-LP-2 1, OLIF-BPS 1, OLIF-NLSLP 1), the osteopenic model (OLIF-SA 2, OLIF-LP-2 2, OLIF-BPS 2, OLIF-NLSLP 2), and the osteoporosis model (OLIF-SA 3, OLIF-LP-2 3, OLIF-BPS 3, OLIF-NLSLP 3). In the Connection interface, define the contact types between each entity. Specifically, the articular surfaces of all facet joints in the model are defined as “non-separation.” The contact surface between the cage and the endplate is characterized by frictional contact with a friction coefficient of 0.8 (22). Additionally, the connections between the screw and the vertebral body, as well as between the screw and the connecting rod, are specified as “bound” (23). Fine meshing was applied to all surgical models, with particular emphasis on ensuring high mesh quality in critical regions such as intervertebral discs and fusion interfaces to enhance analysis accuracy. Specifically, the mesh size for articular cartilage was set to 0.5 mm, while other regions were meshed at 2 mm. The inferior surface of S1 was fixed, and a local coordinate system was established on the superior surface of L3. Based on prior literature, a vertical axial load of 400 N, combined with a radial torque of 7.5 N·m, was applied to simulate six specific physiological motions: flexion, extension, left lateral bending, right lateral bending, left axial rotation, and right axial rotation (14). The numerical values represent the magnitude of the forces, while the plus or minus signs indicate their direction. Analyze and compare the ROM of the L4/5 segments across each OLIF model, as well as the peak von Mises stress values in the endplates, adjacent intervertebral discs, and internal fixation devices.

**Table 1:** Mechanical parameters of spinal model and implants.

Material	Young's Modulus(Mpa)	Poisson's Ratio	Stiffness （N/mm）	References
Cortical bone (NBD)	12000	0.3	-	Liu et al(2023),Yu et al(2024)
Cortical bone (Osteopenia)	10020	0.3	-	Wang et al(2021)
Cortical bone (Osteoporosis)	8,040	0.3	-	Wang et al(2021)
Cancellous bone（NBD）	100	0.2	-	Liu et al(2023),Yu et al(2024)
Cancellous bone（Osteopenia）	67	0.2	-	Wang et al(2021)
Cancellous bone（Osteoporosis）	34	0.2	-	Wang et al(2021)
Posterior element（NBD）	3,500	0.3	-	Liu et al(2023),Yu et al(2024)
Posterior element（Osteopenia）	2,922.5	0.3	-	Wang et al(2021)
Posterior element（Osteoporosis）	2,345	0.3	-	Wang et al(2021)
Endplate（NBD）	2,000	0.2	-	Liu et al(2023),Yu et al(2024)
Endplate（Osteopenia）	1,670	0.2	-	Wang et al(2021)
Endplate（Osteoporosis）	1,340	0.2	-	Wang et al(2021)
Cartilage	25	0.25	-	Liu et al(2023),Yu et al(2024)
Annulus fibrosus	4.2	0.45	-	Liu et al(2023),Yu et al(2024)
Nucleus pulposus	1	0.499	-	Liu et al(2023),Yu et al(2024)
Ligaments				
Anterior longitudinal	-	-	8.74	Kumaran et al(2021),Wang et al(2021)
Posterior longitudinal	-	-	5.83	Kumaran et al(2021),Wang et al(2021)
Transverse ligament	-	-	2.39	Kumaran et al(2021),Wang et al(2021)
Ligamentum flavum	-	-	15.75	Kumaran et al(2021),Wang et al(2021)
Interspinous ligament	-	-	0.19	Kumaran et al(2021),Wang et al(2021)
Supraspinous ligament	-	-	15.38	Kumaran et al(2021),Wang et al(2021)
Cage PEEK	3,600	0.3	-	Yu et al(2024),Li et al(2024)
Spinal instrumentation(titanium alloy)	110,000	0.3	-	Yu et al(2024),Li et al(2024)

## 3 Result

### 3.1 Mesh sensitivity analysis and verification of model validity

According to Ayturk et al. (24), axial rotation is the most sensitive parameter influencing mesh convergence in finite element models. Consequently, this study assessed three mesh sizes under a consistent axial rotational load of 7.5 N·m. Von Mises stress was used as the criterion for assessing mesh convergence, with a difference of less than 5% in results between successive mesh refinements deemed sufficient for convergence (25). Stress values for different tissues across Mesh1 (1 mm), Mesh2 (1.5 mm), and Mesh3 (2 mm) are presented in Table 2. The differences in stress for cortical bone, cancellous bone, nucleus pulposus, and articular cartilage between the mesh configurations were all below the 5% threshold, confirming that the lumbar spine model achieved mesh convergence.

**Table 2:** Mesh sensitivity analysis.

Maximum Mises stresses (Mpa)	Mesh 1 (vs. mesh 2)	Mesh 2	Mesh 3 (vs. mesh 2)
Cortical bone	16.781 (1.02%)	16.611	15.913 (4.20%)
Cancellous bone	0.224 (0.45%)	0.223	0.217 (2.69%)
Nucleus	0.289 (0.35%)	0.288	0.298 (3.47%)
Cartilage	11.344 (4.48%)	10.858	10.983 (1.15%)

**Figure 6:**
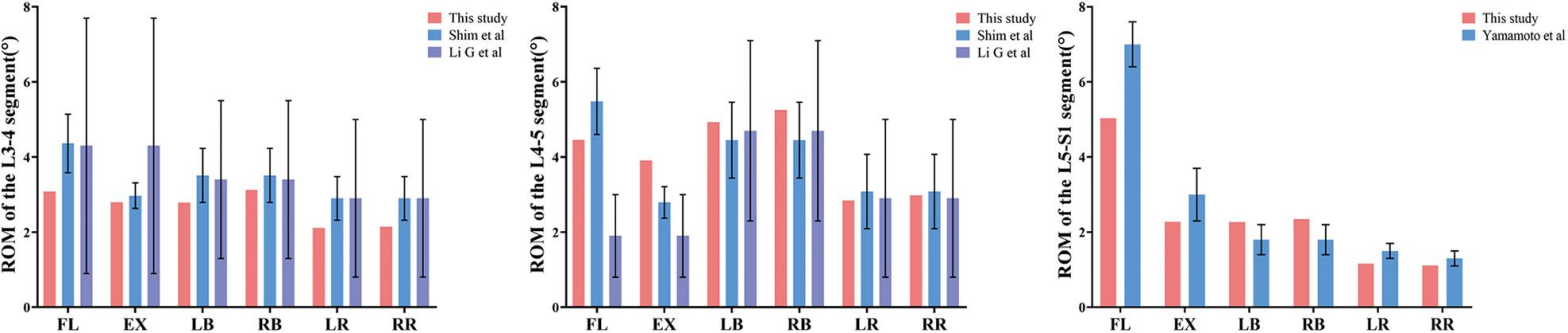
The comparison of the present study on each segment ROM with the cadaveric studies of shim, Li G, and yamamoto in different states of motion.

Prior to conducting the finite element analysis, the three-dimensional finite element model of the intact lumbar spine (L3-S1) was validated. The simulated ROM of the intact model under flexion, extension, lateral bending, and axial rotation closely matched previously reported cadaveric and *in vivo* experimental data (within 10%), confirming the model's validity for subsequent comparative analysis (26–28) Table 3, Figure 6.

**Table 3:** The comparison of the present study on each segment ROM with the cadaveric studies of Shim, Li G, and Yamamoto in different states of motion.

Motion states	L3/4			L4/5			L5/S1	
	This study	Shim, et al	Li G, et al	This study	Shim, et al	Li G, et al	This study	Yamamoto, et al
Flexion	3.08	4.36 ± 0.78	4.3 ± 3.4	4.46	5.48 ± 0.88	1.9 ± 1.1	5.03	7.0 ± 0.6
Extension	2.80	2.97 ± 0.34		3.91	2.79 ± 0.42		2.28	3.0 ± 0.7
Left Bending	2.79	3.51 ± 0.72	3.4 ± 2.1	4.93	4.45 ± 1.01	4.7 ± 2.4	2.27	1.8 ± 0.4
Right Bending	3.13	3.51 ± 0.72		5.25	4.45 ± 1.01		2.35	1.8 ± 0.4
Left Rotation	2.11	2.90 ± 0.58	2.9 ± 2.1	2.84	3.08 ± 0.99	2.9 ± 2.1	1.16	1.5 ± 0.2
Right Rotation	2.14	2.90 ± 0.58		2.98	3.08 ± 0.99		1.11	1.3 ± 0.2

### 3.2 Comparison of the ROM at L4-L5 segment across NLSLP models

**Figure 7:**
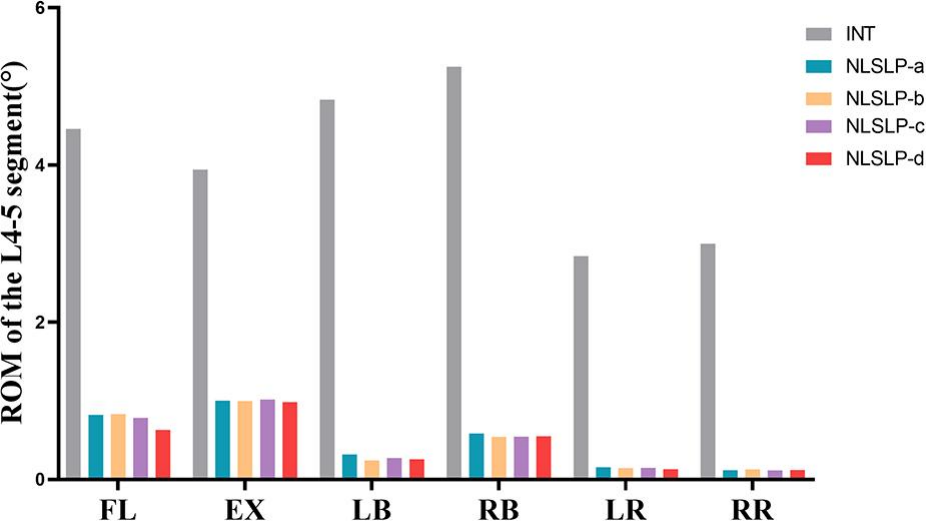
Comparison of the ROM at L4/5 segment across NLSLP models.

Compared with the INT model, all NLSLP configurations exhibited a significant decrease in the ROM of the lumbar spine. For both lateral bending and axial rotation, the ROM of the L4-L5 segment decreased by more than 85% in all NLSLP configurations, accompanied by negligible inter-configuration differences. In flexion and extension, the L4–L5 segmental ROM of the NLSLP-d model was decreased by 85.84% and 75.01%, respectively; this magnitude of reduction was greater than those recorded for the other models. Therefore, NLSLP-d was selected for subsequent comparative analysis. Table 4, Figure 7.

**Table 4:** Comparison of the ROM at L4/5 segment across NLSLP models (unit:°).

Motion states	INT	NLSLP-a	Rates (vs. INT)	NLSLP-b	Rates (vs. INT)	NLSLP-c	Rates (vs. INT)	NLSLP-d	Rates (vs. INT)
Flexion	4.4606	0.8218	−81.58%	0.8325	−84.34%	0.7831	−82.44%	0.6318	−85.84%
Extension	3.9411	1.0026	−74.56%	0.9969	−74.71%	1.0166	−74.21%	0.9849	−75.01%
Left Bending	4.829	0.3184	−93.41%	0.2435	−94.96%	0.2735	−94.34%	0.2585	−94.65%
Right Bending	5.2491	0.5843	−88.87%	0.5434	−89.65%	0.5452	−89.61%	0.5501	−89.52%
Left Rotation	2.8416	0.1571	−94.47%	0.1466	−94.84%	0.1485	−94.77%	0.1313	−95.38%
Right Rotation	2.9982	0.1188	−96.04%	0.1274	−95.75%	0.1155	−96.15%	0.1212	−95.96%

### 3.3 Comparison of the ROM at the L4/5 segment across surgical models

**Figure 8:**
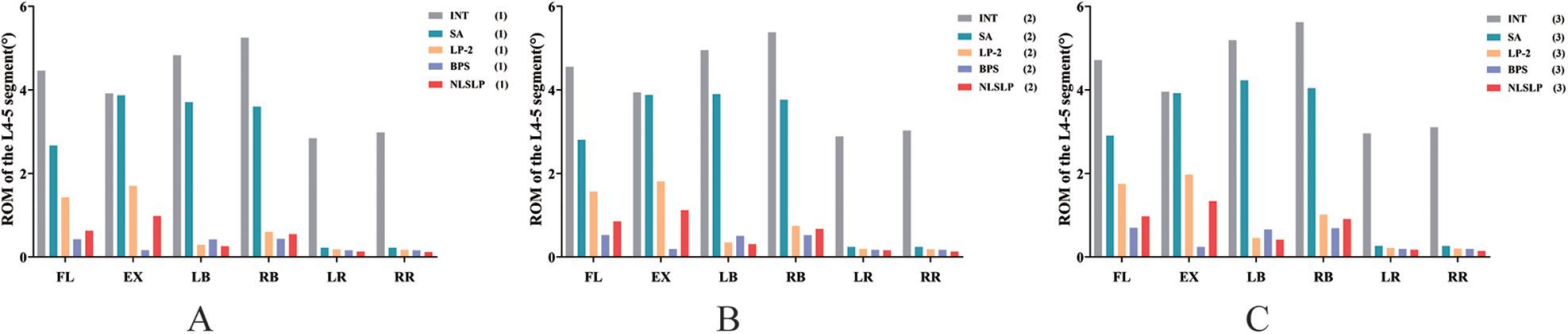
Comparison of L4/5 segmental mobility in different surgical models. **(A)** normal bone density; **(B)** osteopenic; **(C)** osteoporosis.

Relative to the INT model, the L4–L5 segmental ROM was decreased across all surgical configurations. The extent of this reduction was quantified by a standardized percentage reduction, which was defined as: (ROM_surgical_ - ROM_INT_)/ROM_INT_ ×100%. (Table 5, Figure 8.) Among all models, the SA model was associated with the smallest magnitude of ROM reduction, while the BPS model yielded the greatest reduction in L4–L5 segmental mobility.

**Table 5:** Comparison of L4/5 segmental mobility in different surgical models under osteoporotic conditions (group 3) (unit:°).

Motion states	INT	SA	Rates (vs. INT)	LP-2	Rates (vs. INT)	BPS	Rates (vs. INT)	NLSLP	Rates (vs. INT)
Flexion	4.7143	2.9085	−38.30%	1.7522	−62.83%	0.6993	−85.17%	0.9774	−79.27%
Extension	3.9555	3.9237	−0.80%	1.9726	−50.13%	0.2405	−93.92%	1.3379	−66.18%
Left Bending	5.1902	4.2308	−18.48%	0.4524	−91.28%	0.6574	−87.33%	0.4123	−92.06%
Right Bending	5.6197	4.0431	−28.05%	1.0113	−82.00%	0.6883	−87.75%	0.9109	−83.79%
Left Rotation	2.957	0.2665	−90.99%	0.214	−92.76%	0.1936	−93.45%	0.1778	−93.99%
Right Rotation	3.1049	0.264	−91.50%	0.202	−93.49%	0.192	−93.85%	0.1457	−95.31%

#### 3.3.1 Comparison of NLSLP and conventional lateral fixation (LP-2)

In the NBD models, the NLSLP system demonstrated superior restriction of ROM in both flexion and extension compared to the LP-2 system. Specifically, the ROM reduction was 85.84% for flexion and 75.01% for extension with the NLSLP system, compared to 67.99% and 56.79%, respectively, for the LP-2 system. This results in an improvement of 17.85% to 18.22% in controlling sagittal plane motion with the NLSLP system over traditional lateral fixation methods. In terms of lateral bending and axial rotation control, the NLSLP construct showed a modest improvement over the LP-2 system.

#### 3.3.2 Comparison of NLSLP and BPS construct

In the NBD models, the BPS construct achieved comprehensive, well-balanced motion restriction across all anatomical planes, with ROM reductions exceeding 90% in flexion (90.45%), extension (95.86%), lateral bending (91.29%), and axial rotation (94.36%). For the NLSLP construct, the restrictive efficiencies for flexion and extension were 85.84% and 75.01%, respectively, which approached the corresponding values of 95% and 78% achieved by the BPS construct. With regard to lateral bending and axial rotation, the NLSLP and BPS constructs demonstrated no significant differences, and both afforded substantial biomechanical stability.

#### 3.3.3 Effects of osteoporotic conditions on fixation construct performance

In osteoporotic bone models, a universal elevation in L4–L5 segmental ROM was detected across all implanted constructs, signifying diminished global construct stability. Sagittal plane ROM reduction efficiency declined from 85.84% to 79.27% for flexion and from 75.01% to 66.18% for extension in the NLSLP construct; however, motion restriction at the operative level remained above 65% under osteoporotic conditions. Moreover, the flexion and extension restriction efficiencies of NLSLP reached 92.94% and 70.21% of the corresponding values observed in the BPS construct within osteoporotic models. The NLSLP construct retained robust stability in lateral bending and axial rotation even in poor bone quality, exhibiting comparable biomechanical behavior to the BPS construct. Table 5.

### 3.4 Comparison of von mises stress in the intervertebral discs of adjacent segments

**Figure 9:**
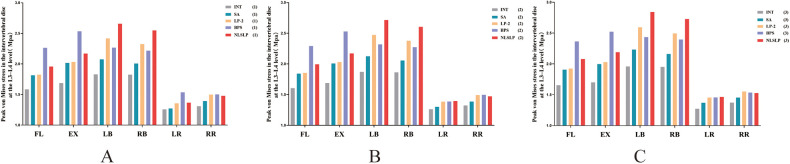
Comparison of von mises stress in the intervertebral disc at the L3–L4 level across different models. **(A)** normal bone density; **(B)** osteopenic; **(C)** osteoporosis.

**Figure 10:**
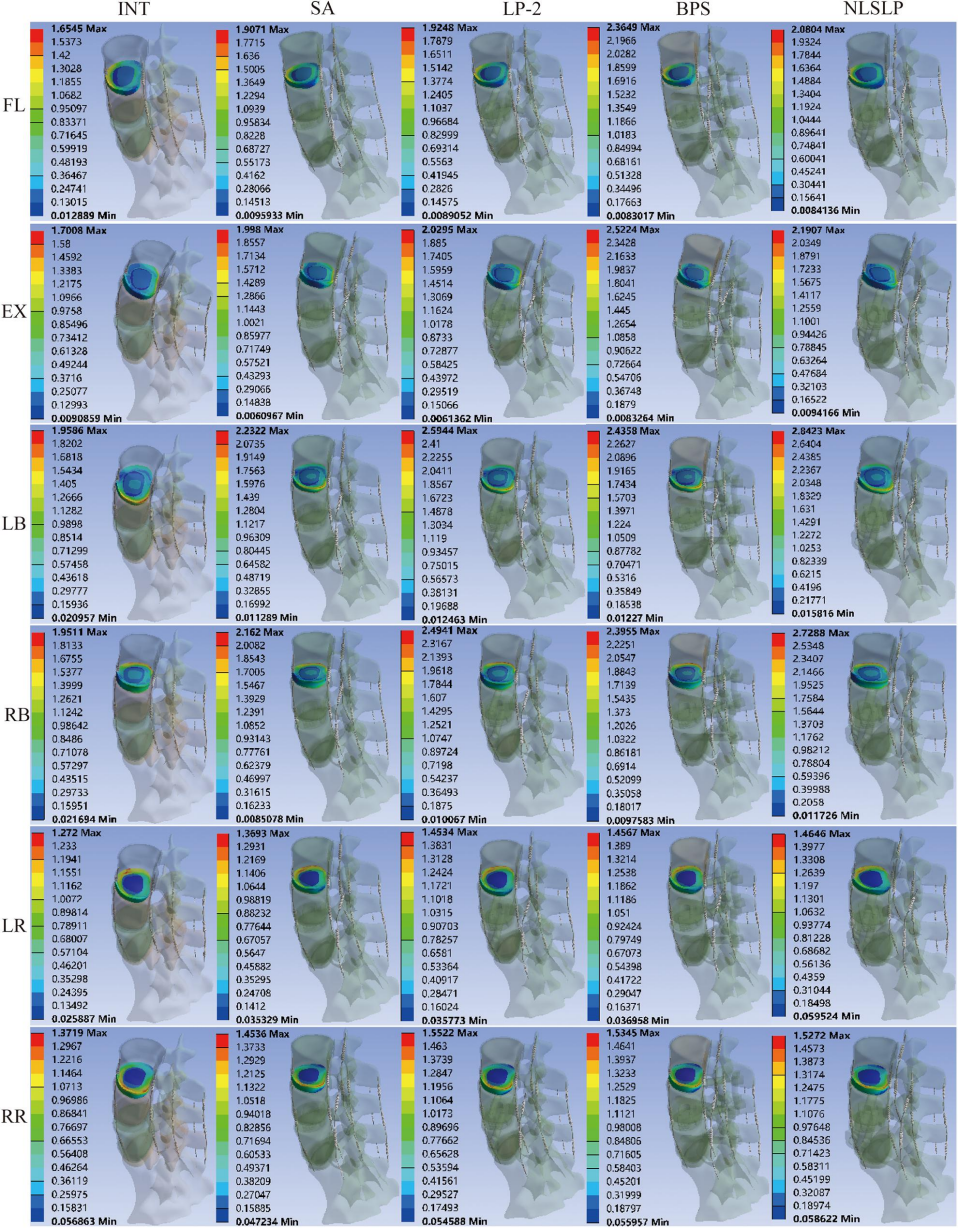
Stress distribution cloud in the L3–L4 intervertebral disc under osteoporotic conditions across all models.

Increased stiffness of the fused segment confers enhanced biomechanical stability, thereby inducing a compensatory elevation in stress within the adjacent intervertebral discs. Figure 9 illustrates the stress comparison of the intervertebral disc at the L3–L4 segment among all models under different bone quality conditions. The results demonstrated that all surgical models induced an increase in the stress of the adjacent-segment intervertebral disc compared with the INT model, and disc stress increased to varying degrees across all models with the progression of osteoporosis. In the osteoporosis group (Group 3) (Table 6), the SA model exhibited the smallest increase in disc stress during flexion–extension, lateral bending, and axial rotation relative to the INT model, with increments of 15.27%–17.47%, 10.81%–13.97%, and 5.96%–7.65%, respectively. In lateral bending, disc stress in the NLSLP model was elevated by 39.86%–45.12%, exceeding the percent increases observed in the LP-2 (27.83%–32.46%) and BPS (22.78%–24.36%) models. For flexion–extension motion, the NLSLP model showed a 25.74%–28.80% rise in disc stress, which was higher relative to the LP-2 model (16.34%–19.33%) yet lower than the BPS model (42.94%–48.31%). Under axial rotation, no significant differences in disc stress were noted between the NLSLP model and either the LP-2 or BPS model. Stress distribution cloud in the L3–L4 intervertebral discs across all models under osteoporosis conditions is presented in Figure 10.

**Table 6:** Comparison of von mises stress in the intervertebral disc at the L3–L4 level across different models under osteoporotic conditions (group 3) (unit: MPa).

Motion states	INT	SA	Rates (vs. INT)	LP−2	Rates (vs. INT)	BPS	Rates (vs. INT)	NLSLP	Rates (vs. INT)
Flexion	1.6545	1.9071	15.27%	1.9248	16.34%	2.3649	42.94%	2.0804	25.74%
Extension	1.7008	1.998	17.47%	2.0295	19.33%	2.5224	48.31%	2.1907	28.80%
Left Bending	1.9586	2.2322	13.97%	2.5944	32.46%	2.4358	24.36%	2.8423	45.12%
Right Bending	1.9511	2.162	10.81%	2.4941	27.83%	2.3955	22.78%	2.7288	39.86%
Left Rotation	1.272	1.3693	7.65%	1.4534	14.26%	1.4567	14.52%	1.4646	15.14%
Right Rotation	1.3719	1.4536	5.96%	1.5522	13.14%	1.5345	11.85%	1.5272	11.32%

### 3.5 Comparison of endplate stress at the surgical segment across different models

**Figure 11:**
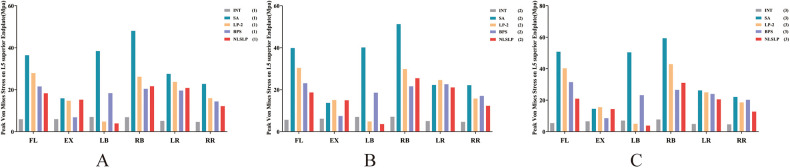
The comparison of the stress on the superior endplate of L5 in different models. **(A)** normal bone density; **(B)** osteopenic; **(C)** osteoporosis.

**Figure 12:**
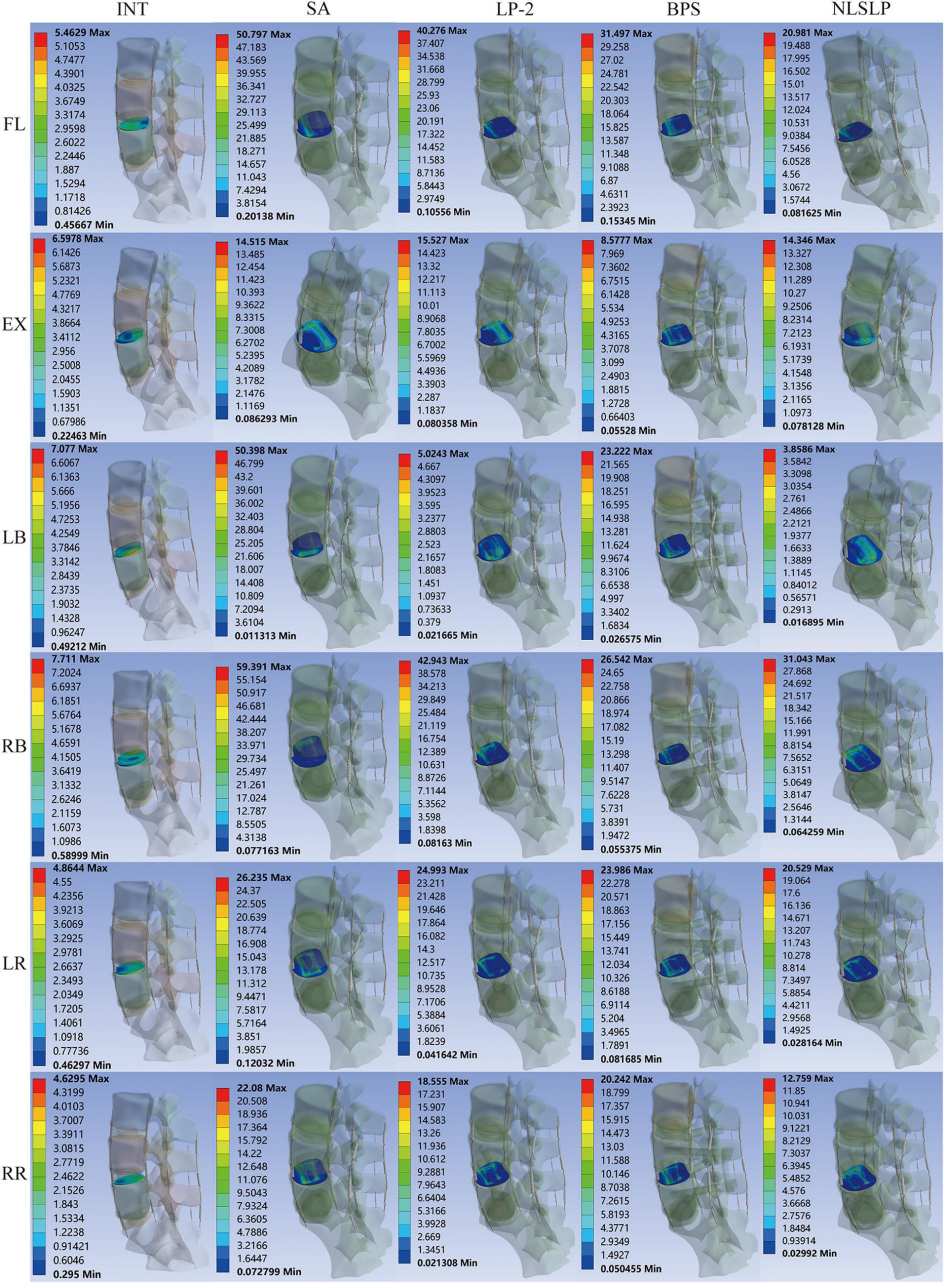
Stress distribution cloud on the L5 superior endplate under osteoporotic conditions across all models.

Figure 11 illustrates the comparison of L5 superior endplate stress across all models under varying bone quality conditions. In Group 1 (NBD), the SA model showed a significant 166.12%–597.18% increase in L5 superior endplate stress compared with the INT model. By contrast, the NLSLP, LP-2, and BPS models with auxiliary internal fixation significantly reduced endplate stress relative to the SA model. As osteoporosis progressed, L5 superior endplate stress exhibited a gradual increase across all surgical models under most loading conditions. Within the osteoporotic cohort (Group 3) (Table 7), the NLSLP construct reduced L5 superior endplate stress by 58.70%, 92.34%, 21.75%, and 42.21% in flexion, left lateral bending, left axial rotation, and right axial rotation, respectively, relative to the SA model. This stress-mitigating effect outperformed both the BPS (38.03%, 53.92%, 8.57%, and 8.32%) and LP-2 (20.71%, 90.03%, 4.73%, and 15.96%) constructs. Nevertheless, the stress reduction of the NLSLP construct during extension was merely 1.16%, which was markedly lower than the 40.90% reduction attained by the BPS construct. Of note, a 6.97% elevation in L5 superior endplate stress was observed in the LP-2 model as compared with the SA model. Stress distribution cloud in the L5 superior endplate across all models under osteoporosis conditions is presented in Figure 12.

**Table 7:** The comparison of the stress on the superior endplate of L5 in different models under osteoporotic conditions (group 3) (unit: Mpa).

Motion states	SA	LP-2	Rates (vs. SA)	BPS	Rates (vs. SA)	NLSLP	Rates (vs. SA)
Flexion	50.797	40.276	−20.71%	31.479	−38.03%	20.981	−58.70%
Extension	14.515	15.527	6.97%	8.5777	−40.90%	14.346	−1.16%
Left Bending	50.398	5.0243	−90.03%	23.222	−53.92%	3.8586	−92.34%
Right Bending	59.391	42.943	−27.69%	26.542	−55.31%	31.043	−47.73%
Left Rotation	26.235	24.993	−4.73%	23.986	−8.57%	20.529	−21.75%
Right Rotation	22.08	18.555	−15.96%	20.242	−8.32%	12.759	−42.21%

### 3.6 Comparison of internal fixation stress across different models

**Figure 13:**
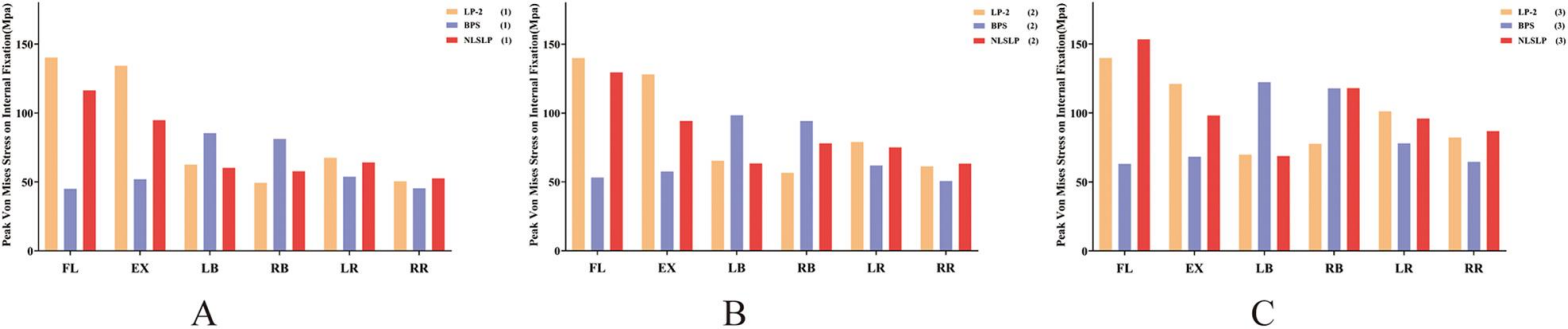
Comparison of internal fixation stress across different models. **(A)** normal bone density; **(B)** osteopenic; **(C)** osteoporosis.

**Figure 14:**
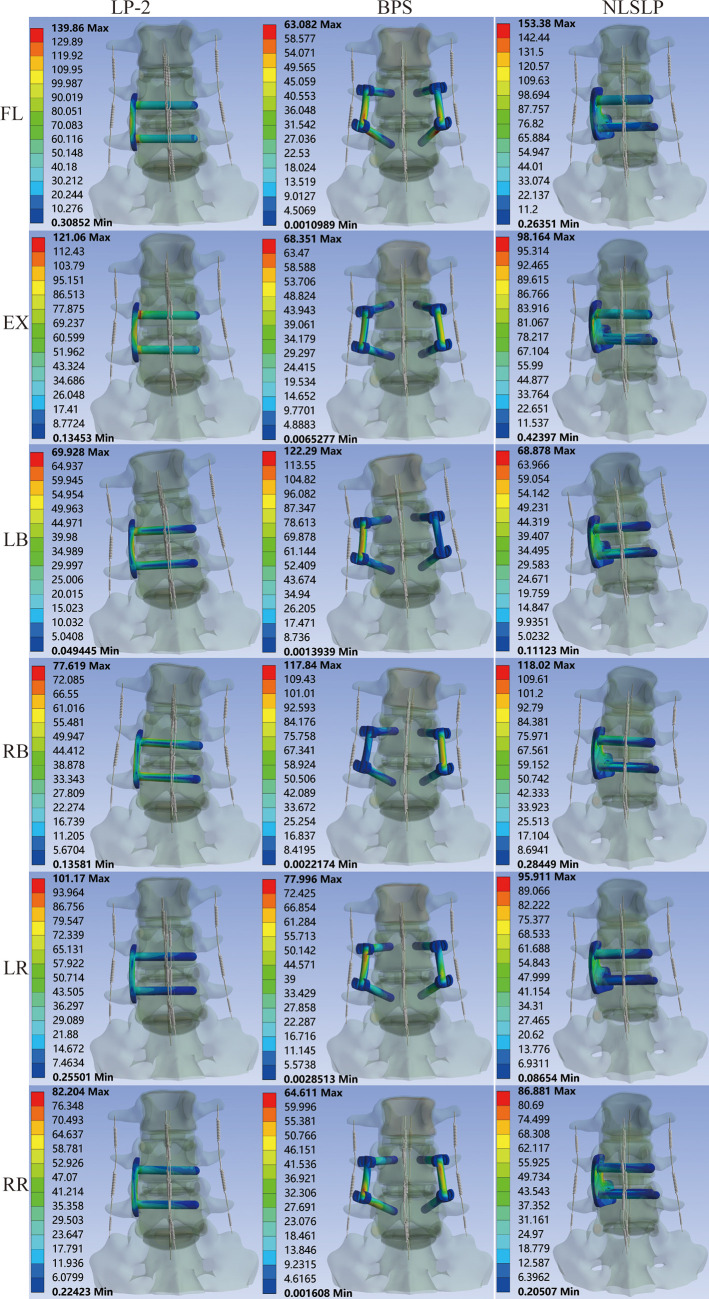
Stress distribution cloud on the internal fixation under osteoporotic conditions across all models.

The peak stress on the internal fixation across different models was evaluated (Table 8 and Figure 13) and compared to the fatigue strength (310–610 MPa) and yield strength (789–1,013 MPa) of the Ti-6Al-4 V alloy (29). In NBD model, the NLSLP system exhibited favorable stress distribution under most loading conditions. During flexion and extension, stress values for the NLSLP system were lower than those of the LP-2 model but higher than those of the BPS model. Under left and right bending, stresses in the NLSLP system were comparable to those of the LP-2 model and lower than those of the BPS model. With increasing osteoporosis severity, stresses in the NLSLP and BPS increased, whereas LP-2 showed increased stress during lateral bending and rotation but slight decreases during flexion and extension. Specifically, NLSLP stress increased by 3.54%–104.38%, and BPS by 31.50%–45.08%. For LP-2, stress increased by 11.76%–62.68% during lateral bending and rotation but decreased by 0.26%–9.90% during flexion and extension. Despite these increases, the maximum stress in NLSLP (153.38 MPa) remained 50.52% below the fatigue strength lower bound (310 MPa) and 80.56% below the minimum yield strength (789 MPa) of the Ti-6Al-4 V alloy. Stress distribution cloud on the internal fixation across all models is presented in Figure 14.

**Table 8:** Comparison of internal fixation stress across different models (unit: Mpa).

Motion states	Flexion	Extension	Left Bending	Right Bending	Left Rotation	Right Rotation
LP-2						
NBD	140.22	134.36	62.568	49.409	67.551	50.53
Osteopenia	139.98	128.11	65.377	56.605	79.054	61.244
Osteoporosis	139.86	121.06	69.928	77.619	101.17	82.204
BPS						
NBD	44.971	51.956	85.487	81.223	53.83	45.387
Osteopenia	53.238	57.575	98.407	94.209	61.986	50.669
Osteoporosis	63.082	68.351	122.29	117.84	77.996	64.611
NLSLP						
NBD	116.4	94.292	60.279	57.746	64.198	52.65
Osteopenia	129.5	94.804	63.501	77.969	75.032	63.305
Osteoporosis	153.38	98.164	68.878	118.02	95.911	86.881

## 4 Discussion

The selection of an optimal supplemental fixation strategy for OLIF requires balancing sufficient biomechanical stability with preservation of the minimally invasive advantages inherent to the procedure. Supplemental fixation serves two primary biomechanical functions: enhancing segmental stability to facilitate successful interbody fusion and optimizing load distribution across the vertebral endplates to reduce the risk of cage subsidence (30). In the present study, we developed a NLSLP and systematically evaluated its biomechanical performance using validated finite element models under varying bone density conditions.

Our validated lumbar finite element model demonstrated strong agreement with previously published cadaveric and *in vivo* biomechanical data, supporting its reliability for comparative analysis (26–28). Consistent with prior studies, BPS fixation provided the greatest restriction of motion across all loading directions, confirming its established role as the biomechanical reference standard for spinal stabilization. BPS reduced ROM by more than 90% in all motion planes, reflecting its capacity to create a highly rigid fixation construct. However, despite its superior stability, BPS fixation compromises several key advantages of OLIF, including single-position surgery, minimal soft tissue disruption, and reduced operative morbidity. The requirement for posterior instrumentation increases operative time, soft tissue injury, and radiation exposure, and may contribute to accelerated adjacent segment degeneration due to excessive construct stiffness (31).

In contrast, lateral fixation techniques have emerged as less invasive alternatives that preserve the minimally invasive philosophy of OLIF. Conventional two-screw lateral plate fixation (LP-2) improves stability compared to stand-alone OLIF but demonstrates limited effectiveness in controlling sagittal plane motion (11). Previous *in vitro* studies reported that lateral plate fixation reduced flexion-extension ROM by approximately 49.5% (32). Our findings are consistent with these results, showing sagittal plane motion reductions of 50.13–62.83% with LP-2 fixation. However, this level of stabilization may be insufficient, particularly in osteoporotic bone, where compromised bone quality further reduces fixation strength and increases the risk of cage subsidence.

To address this limitation, the NLSLP was designed based on biomechanical principles emphasizing bicortical fixation and spatial triangular screw configuration. Our results demonstrated that the NLSLP significantly improved sagittal plane stability compared with LP-2 fixation. In the normal bone density model, the NLSLP reduced ROM by 85.84% in flexion and 75.01% in extension, representing improvements of approximately 18% compared to LP-2 fixation. Importantly, even under osteoporotic conditions, the NLSLP maintained greater than 65% motion restriction, indicating preserved fixation effectiveness despite reduced bone stiffness.

The improved biomechanical performance of the NLSLP is primarily attributable to its geometric configuration and screw orientation. The L-shaped plate and triangular screw arrangement enhance resistance to multidirectional loading by increasing construct rigidity and improving force distribution. During lateral bending and axial rotation, the screw axes are oriented perpendicular to the rotational axis, creating a longer moment arm that enhances resistance to motion. In contrast, during flexion and extension, the lateral fixation construct provides relatively less mechanical leverage compared to posterior fixation, explaining why BPS fixation still demonstrated superior sagittal stability. Nevertheless, the NLSLP achieved sagittal plane stability approaching that of BPS while preserving the minimally invasive advantages of lateral fixation.

Adjacent segment biomechanics represent an important consideration following spinal fusion. Increased stress in adjacent intervertebral discs reflects compensatory load transfer resulting from reduced motion at the fused segment (33). Our results showed increased adjacent disc stress across all fixation constructs, with the magnitude of stress elevation correlating with construct rigidity. The NLSLP produced greater adjacent disc stress than LP-2 fixation but less than BPS fixation during flexion and extension. This intermediate stress profile suggests that the NLSLP achieves an effective balance between stability and physiological load sharing, potentially reducing the risk of excessive adjacent segment degeneration associated with overly rigid constructs.

Endplate stress analysis provides critical insight into the risk of cage subsidence, particularly in osteoporotic bone. The stand-alone OLIF model predictably results in significant stress concentration at the endplate, with stress increases exceeding 166.11% across most movements. In osteoporotic conditions, the maximum increase reached 829%, highlighting the clinical risk of cage subsidence as reported by Hu et al. (5) Therefore, stand-alone OLIF is not recommended for patients with osteoporosis. After the application of the auxiliary internal fixation device, there was a significant reduction in stress on the endplate. Interestingly, under osteoporotic conditions, the LP-2 model showed an unexpected increase in stress on the superior endplate of L5 compared to the SA model. This may be one of the reasons for the increased risk of cage subsidence associated with LP-2, due to its insufficient sagittal plane stability. Chen et al. (34) reported that there was no significant difference in cage subsidence between lateral interbody fusion with and without supplemental LP-2 fixation. Some researchers suggest that LP-2 fixation may elevate the risk of intervertebral cage subsidence and vertebral fracture, possibly due to screw-induced disruption of the trabecular architecture in the subchondral bone (35, 36). In contrast, the NLSLP system consistently reduces endplate stress under all loading conditions. However, in the osteoporotic group (Group 3), the reduction in endplate stress during extension was limited to only 1.16%, significantly lower than that observed with the BPS system. This diminished stress reduction suggests a decline in the biomechanical stability of the NLSLP system under extension loading, likely due to the intrinsic limitations of lateral fixation constructs. Clinically, patients with osteoporosis who undergo NLSLP implantation may benefit from limiting excessive backward extension and using a lumbar orthosis postoperatively to counteract this relative instability.

Implant stress analysis further demonstrated favorable biomechanical performance of the NLSLP. In the osteoporotic model, peak stress on the NLSLP system occurred during flexion (153.38 MPa), well below the fatigue strength (310 MPa) and yield strength (789 MPa) of the Ti6Al4 V alloy (29). This suggests a low risk of implant failure under physiological loading conditions. However, fatigue strength refers to the maximum stress a material can withstand under repeated cyclic loading, which differs from the stress limit observed under a single static load. Therefore, this can only provide preliminary biomechanical guidance for clinicians.

Given that the fixation positions of the NLSLP may influence stability in the surgical segment, four finite element models were developed for analysis and comparison. Compared to the INT model, all NLSLP models significantly reduced the ROM in the surgical segment. Considering the minimal differences in lateral bending and axial rotation among the models, the optimal configuration was determined based on performance in the sagittal plane. The NLSLP-d configuration demonstrated the greatest restriction in both flexion (85.84%) and extension (75.01%), outperforming other NLSLP models. The biomechanical advantage of the NLSLP-d configuration in flexion-extension is attributed to its strategic screw placement. The cephalad screw is positioned near the posterior margin of the vertebral body, and, along with the anteriorly placed OLIF cage and two caudad screws, forms an effective four-point support structure. This setup provides an elongated moment arm, significantly enhancing resistance to moments in the sagittal plane. Therefore, the NLSLP-d method is recommended for fixation in OLIF procedures to ensure surgical stability and optimize postoperative outcomes.

Limitations of this study: Firstly, the finite element model typically requires simplification or omission of complex structures in the lumbar vertebrae (e.g., intervertebral discs, cartilage endplates, ligaments, and muscles), potentially resulting in inaccuracies in mechanical load transmission simulations. Secondly, the lumbar spine tissues—including cortical bone, cancellous bone, intervertebral discs, and ligaments—are modeled as linear elastic and isotropic materials. Nevertheless, the mechanical behavior of human lumbar vertebrae specimens is not simply that of a linear material, but rather exhibits significant nonlinear characteristics. Variations in material properties may lead to deviations in the experimental results. Thirdly, the assessment of implant stress and fatigue risk in this study was based on static peak stress under single-loading conditions. In clinical practice, fatigue failure of spinal implants is predominantly related to cyclic loading over time. Fourthly, the finite element model was established based on CT data from a typical patient, involving a small sample size. This limitation is inherent in finite element studies. In future work, *in vitro* specimen experiments will be conducted to further validate and analyze the biomechanical stability of the system.

## 5 Conclusions

The NLSLP represents a viable adjunctive fixation option for OLIF, offering superior sagittal plane stability compared to LP-2 while retaining the minimally invasive and single-position advantages of the OLIF procedure.

## Data Availability

The original contributions presented in the study are included in the article/Supplementary Material, further inquiries can be directed to the corresponding author.
